# Egg Yolk Antibodies Elicited by a Novel Multi-Epitope Recombinant Adenovirus Vaccine Against Genotype G2b PEDV Spike Protein Reduce Mortality and Viral Shedding in Passively Immunized Piglets

**DOI:** 10.3390/pathogens15060602

**Published:** 2026-06-03

**Authors:** Cunyi Qiu, Zhiding Zhou, Meilin Yang, Huaxin Wang, Xuezhao Li, Zhihua Feng, Yefei Zhou

**Affiliations:** 1 Department of Food Science, Nanjing Xiaozhuang University, Nanjing 211171, China; vetqcy5495@nwafu.edu.cn (C.Q.); 17309367693@163.com (M.Y.); 2 College of Veterinary Science and Technology, Gansu Polytechnic College of Animal Husbandry & Engineering, Wuwei 733006, China; wanghuaxin2021@163.com (H.W.); lixuezhao621@163.com (X.L.); zhihuaf@163.com (Z.F.); 3 College of Veterinary Medicine, Sichuan Agricultural University, Chengdu 611130, China; zhidingzhou@163.com

**Keywords:** porcine epidemic diarrhea virus (PEDV), adenoviral vector vaccine, egg yolk antibodies (IgY), piglets

## Abstract

Porcine epidemic diarrhea (PED), caused by the PED virus (PEDV), remains one of the most devastating diseases in the swine industry, with a mortality rate approaching 90–100% in suckling piglets due to severe dehydration and electrolyte imbalances. Passive immunization with egg yolk antibodies (IgY) represents a promising therapeutic strategy. In this study, we developed a novel recombinant adenovirus, rADM-IFN-G-ped, co-expressing selected antigenic regions of the PEDV S protein and chicken interferon-gamma (ChIFN-γ) as a molecular adjuvant. Laying hens were immunized with this construct to produce PEDV-specific IgY, which was subsequently purified from eggs using a polyethylene glycol (PEG-6000) precipitation method. The induced IgY demonstrated potent neutralizing activity against PEDV in vitro, with a neutralization titer (NT_50_) of 1:96, which was significantly higher than that of IgY derived from hens immunized with a commercial inactivated PEDV G2b vaccine (NT_50_ = 1:52). In a passive immunization and challenge trial, piglets treated with the rADM-IFN-G-ped-derived IgY exhibited significantly reduced fecal viral RNA shedding following challenge with the virulent PEDV-NX-2022 strain, compared to control groups. Crucially, while all piglets in the challenge control group succumbed to infection within 72 h, a 50% survival rate was achieved in the IgY-treated group. Histopathological examination of intestinal tissues further confirmed the protective efficacy, showing that IgY treatment markedly alleviated villous atrophy, epithelial necrosis, and inflammatory cell infiltration in the small intestine. These findings demonstrate that vaccination of laying hens with the rADM-IFN-G-ped recombinant adenovirus elicits a robust immune response, enabling the production of protective IgY. This proof-of-concept study establishes the viability of the multi-epitope adenoviral IgY platform as a passive immunization strategy against PEDV.

## 1. Introduction

Porcine epidemic diarrhea (PED), caused by the porcine epidemic diarrhea virus (PEDV), is one of the most economically devastating diseases in the global swine industry. Clinical signs include acute diarrhea, vomiting, and dehydration, with mortality rates approaching 90–100% in neonatal piglets. This high susceptibility is attributed to their underdeveloped immune systems and poor ability to compensate for severe fluid and electrolyte loss. Secretory immunoglobulin A (sIgA) plays a pivotal role in mucosal immunity against enteric pathogens like PEDV. It is transported across mucosal epithelia and secreted into colostrum, providing crucial passive immunity (lactogenic immunity) to suckling piglets. Consequently, the primary control strategy in sow herds involves vaccinating pregnant sows with inactivated or live-attenuated PEDV vaccines to stimulate the production of milk-borne sIgA [1,2,3]. However, conventional parenteral (e.g., intramuscular) vaccination often fails to induce robust mucosal immune responses in sows [4,5]. This can lead to insufficient sIgA transfer via the gut-mammary-sIgA axis, leaving piglets vulnerable to intestinal viral replication, severe diarrhea, and death [6,7]. Furthermore, the continuous emergence of PEDV variants with genetic drift may compromise the efficacy of existing commercial vaccines [8]. These limitations underscore the need for alternative strategies, such as direct passive immunization of piglets via oral administration of pathogen-specific antibodies.

IgY have emerged as a promising tool for passive immunization. They can be produced cost-effectively at scale by immunizing laying hens and have proven effective against various enteric pathogens [9]. Studies have demonstrated that IgY specific to PEDV, generated by immunizing hens with inactivated virus or recombinant S1 protein, can confer significant protection to challenged piglets [10,11]. This establishes IgY as a viable candidate for a passive immunoprophylaxis strategy against PED.

Adenovirus serotype 5 (Ad5) vectors have been successfully employed in the development of numerous veterinary and human vaccines due to their favorable properties [12,13]. These include a well-characterized genome, high transduction efficiency across diverse cell types (including avian and mammalian), robust production of high-titer stocks, and a strong capacity to induce both humoral and cellular immune responses [12]. Importantly, Ad5 vectors are non-replicating and non-integrating, offering a strong safety profile. Notably, recombinant Ad5 vectors have shown promise in inducing mucosal immunity, a critical feature for vaccines targeting enteric diseases. Their proven efficacy in vaccines against pseudorabies [14,15], classical swine fever [16], and influenza highlights [17,18] their broad potential as a vaccine platform. The PEDV spike (S) glycoprotein is responsible for viral attachment and entry and is the primary target for neutralizing antibodies, making it the ideal antigen for vaccine design [19]. Various recombinant vaccines expressing the S protein or its domains—including lactic acid bacteria [20], attenuated Salmonella [21], and parapoxvirus [22] vectors—have been explored. The first conditionally licensed PEDV vaccine in the United States was also an S-protein-based replicon vaccine [23]. This collective evidence solidifies the S protein as the key target for subunit and vectored vaccines.

Currently available commercial PEDV vaccines are mostly developed based on classical strains or early variants and have significant limitations: conventional inactivated vaccines contain low effective antigen content and fail to induce robust mucosal or cellular immunity [8]; live attenuated vaccines, although capable of eliciting high-titer neutralizing antibodies, carry the risk of virulence reversion and recombination with field strains [24]. An even greater challenge is that the S protein, as the key antigenic target, undergoes frequent mutations, leading to a mismatch between vaccine strains and circulating GII genotype strains [25], such that vaccine development lags far behind viral evolution. To address the current challenges in PED control, this study designed a novel recombinant Ad5 vaccine (rADM-IFN-G-ped) expressing a concatenated construct of four key neutralizing epitope regions (aa 19–44, 54–146, 298–315, and 475–774) from the S protein of a prevalent G2b strain, PEDV-NX-2022. This multi-epitope antigen was co-expressed with chicken interferon-gamma (ChIFN-γ), a molecular adjuvant expected to enhance overall immunogenicity by promoting antigen presentation and T-cell responses. Relevant studies have shown that multi-epitope vaccines offer a theoretically promising avenue for establishing durable immunity against pathogens [26]. Multi-epitope vaccines have demonstrated efficacy against a variety of viruses, including influenza A [27], hemorrhagic fever [28], Ebola [29], and hepatitis C [30]. Beyond excluding undesirable antigens, multi-epitope vaccines offer additional advantages over conventional vaccines because they integrate multiple proteins with distinct biological functions, thereby more effectively activating cellular and humoral immune responses [31]. Based on this rationale, we hypothesized that immunizing laying hens with this recombinant adenovirus would elicit high-titer neutralizing IgY. Subsequent purification of this IgY and its oral administration to piglets was proposed as an effective passive immunization strategy to reduce mortality and viral shedding following PEDV challenge. This approach aims to bypass the limitations of lactogenic immunity and provide direct, immediate protection to the most vulnerable population—newborn piglets. It is important to note that this study was designed as a proof-of-concept to validate the overall platform; it does not include direct comparative data to quantify the specific contribution of the multi-epitope format over full-length or single-domain antigens.

## 2. Materials and Methods

### 2.1. Virus, Cells and Experimental Animals

The virulent PEDV G2b strain PEDV-NX-2022 (GenBank accession: PZ161424) was isolated and maintained in our laboratory. HEK 293 cells and the AdMax adenoviral system were sourced from Vigene Biosciences (Jinan, China). Vero cells were kindly gifted by Professor Zheng Hongqing of Xianyang Vocational College. HEK 293 cells (ATCC^®^ CRL-1573™) and Vero cells (ATCC^®^ CCL-81™) were cultured in DMEM (Gibco, Grand Island, NY, USA) supplemented with 10% FBS (Gibco, USA), 100 U/mL penicillin, and 100 µg/mL streptomycin at 37 °C in a 5% CO_2_ incubator. Sixty, 25-week-old Hy-Line Brown laying hens were purchased from Nanjing Poultry Institute Hatchery (Nanjing, China). Hens were housed in individual cages under controlled conditions with ad libitum access to food and water. Eighteen, 4-day-old crossbred (Duroc × Landrace × Yorkshire) piglets, PEDV-naïve as confirmed by PCR, were obtained from a commercial farm (Nanjing Lukang Pig Farm, Nanjing, China) with a well-documented history of no PEDV outbreaks. Piglets were randomly allocated and housed in separate, HEPA-filtered isolation units within a biosafety level 2 facility. All animal experiments were conducted in strict accordance with the guidelines of the Nanjing Xiaozhuang University Animal Care and Use Committee. The protocol was approved by the Committee (Approval No. IACECNXU20250725).

### 2.2. Construction and Preparation of Recombinant Adenovirus

The construction of the recombinant adenovirus was initiated by designing a multi-epitope antigen. Key neutralizing epitope regions within the S protein of the PEDV G2b isolate PEDV-NX-2022 were identified through bioinformatic analysis using the Protean module of DNASTAR software 11.0 and corroborated by published literature on PEDV B-cell epitopes. Four critical regions (aa 19–44, 54–146, 298–315, and 475–774) were selected. These epitope sequences were concatenated in silico using flexible (GGGGS)_3_ linkers. To enhance the immunogenicity of the vaccine candidate, the designed multi-epitope fragment was fused upstream of the ChIFN-γ gene via a P2A self-cleaving peptide linker, which ensures the independent translation of both proteins from a single bicistronic mRNA. The final construct, optimized for chicken-preferred codon usage, was synthesized de novo and cloned into the pUC57 vector by Tsingke Biotech Co., Ltd. (Xi’an, China). For adenovirus generation, the synthesized gene fragment was excised and subcloned into the *AsiSI* and *MluI* restriction sites of the adenoviral shuttle plasmid pADM-CMV-C-FH using T4 DNA ligase, generating the recombinant shuttle plasmid pADM-CMV-C-FH-IFN-G-ped. Correct plasmid construction was confirmed by restriction enzyme digestion with *AsiSI* and *MluI* and subsequent analysis by 1% agarose gel electrophoresis. Recombinant adenovirus was rescued by co-transfecting linearized pADM-CMV-C-FH-IFN-G-ped shuttle plasmid with the adenoviral backbone plasmid pAdeasy-1 into HEK 293 cells using Lipofectamine 3000 reagent (Invitrogen, Carlsbad, CA, USA), following the manufacturer’s instructions. Cells were monitored for the development of a CPE. The primary viral stock, designated rADM-IFN-G-ped, was harvested by three cycles of freeze–thaw lysis. The virus was then subjected to three rounds of plaque purification on HEK 293 cells to obtain a clonally purified stock. The purified virus was subsequently amplified in HEK 293 cells, and high-titer viral stocks were prepared and concentrated by CsCl density gradient ultracentrifugation. The infectious titer of the final purified rADM-IFN-G-ped stock was determined using a TCID_50_ assay on HEK 293 cells and is expressed as TCID_50_/mL.

### 2.3. In Vitro Verification of Recombinant Protein Expression

Expression of the PEDV S protein epitopes by the recombinant adenovirus was confirmed using two complementary immunological methods: Western blot and indirect immunofluorescence assay (IFA). For Western blot analysis, HEK 293 cells infected with rADM-IFN-G-ped or the negative control adenovirus rADM-blank were harvested 24 h post-infection. Cells were washed twice with ice-cold PBS and lysed in RIPA buffer (Beyotime, Shanghai, China) containing protease inhibitor cocktail (MedChemExpress, Monmouth Junction, NJ, USA) on ice for 30 min. The lysates were centrifuged at 12,000× *g* for 15 min at 4 °C, and the supernatant was collected. Protein concentration was determined using a BCA Protein Assay Kit (Beyotime, Shanghai, China). The protein concentration of each sample was adjusted to 5 μg/μL using loading buffer. Equal amounts of protein (20 μg per lane) were denatured, separated by 10% SDS-PAGE, and transferred onto a PVDF membrane (Tsingke Biotech Co., Ltd., Xi’an, China). The membrane was then cut into strips corresponding to the molecular weights of the target protein and the loading control. One strip was incubated overnight at 4 °C with a mouse anti-PEDV S protein monoclonal antibody (1:1500 dilution; kindly provided by Prof. Zheng Hongqing, Xianyang Vocational College, Xianyang, China). Another strip was incubated with a mouse anti-β-tubulin monoclonal antibody (1:2000 dilution; Abmart, Shanghai, China) as a loading control. After washing, both strips were incubated with the same horseradish peroxidase (HRP)-conjugated rabbit anti-mouse IgG Fc secondary antibody (1:5000 dilution; Abmart, Shanghai, China) for 1 h at room temperature. Specific protein bands were visualized using an ECL substrate (MedChemExpress, USA). For the IFA, HEK 293 cells grown on coverslips were infected with rADM-IFN-G-ped at an MOI of 5. At 48 h post-infection, cells were fixed with 4% paraformaldehyde, permeabilized with 0.1% Triton X-100 (MedChemExpress, USA), and blocked. The cells were then incubated with the same mouse anti-PEDV S protein monoclonal antibody (1:1500 dilution) used for Western blot, followed by a FITC-conjugated goat anti-mouse IgG secondary antibody. Cell nuclei were counterstained with DAPI (4′,6-diamidino-2-phenylindole). Fluorescent signals were examined and imaged using an Olympus IX73 inverted fluorescence microscope (Olympus Corporation, Tokyo, Japan) equipped with a 20× objective lens and a DP80 CCD camera. Uninfected HEK 293 cells and cells infected with a control adenovirus (rADM-blank) were processed in parallel as negative controls.

### 2.4. Immunization of Laying Hens and Antibody Level Monitoring

Sixty 25-week-old Hy-Line Brown commercial laying hens were randomly divided into three groups (n = 20 per group). Group 1 was immunized with the recombinant adenovirus vaccine rADM-IFN-G-ped. Group 2 received a negative control adenovirus, rADM-blank. Group 3 was immunized with a commercially available inactivated PEDV vaccine (Keqian Biology, Wuhan, China) as a positive control. All hens were immunized via intramuscular injection into the pectoral muscle with a dose of 1 × 10^8^ TCID_50_ per bird per immunization. A prime-boost immunization regimen was administered three times at 0, 14, and 28 days. To monitor the dynamic changes in specific IgY antibody levels, blood and egg samples were collected at scheduled time points (0, 14, 28, 35, 42, and 49 days post-initial immunization). At each time point, six hens were randomly selected from each group for blood collection (approximately 1 mL per hen) from the wing vein. Blood samples were allowed to clot and were then centrifuged at 12,000× *g* rpm for 10 min at 4 °C to separate the serum, which was aliquoted and stored at −20 °C. Concurrently, six eggs were collected from each group at the same time points, and yolk antibodies (IgY) were purified following the method described in Section 2.5 and stored at −20 °C.

The titers of PEDV-specific IgY antibodies in serum and purified yolk samples were determined using an indirect ELISA. A 96-well high-binding ELISA plate (Corning, NY, USA) was coated with purified whole PEDV viral particles (5 μg/mL in carbonate-bicarbonate coating buffer, 0.05 M, pH 9.6; Solarbio, Beijing, China) at 100 μL/well overnight at 4 °C. After washing three times with PBST (PBS containing 0.05% Tween-20), plates were blocked with 1% bovine serum albumin (BSA) in PBST for 1 h at 37 °C. For kinetic monitoring, based on preliminary experiments, serum samples were diluted 1:400 and purified IgY samples were diluted 1:200 in PBST containing 1% BSA to ensure OD_450_ values within the linear range. For endpoint titer determination, serial two-fold dilutions were prepared (starting from 1:100 for serum and 1:50 for IgY). Following sample incubation, a horseradish peroxidase (HRP)-conjugated goat anti-chicken IgY secondary antibody (Abmart, Shanghai, China) was added. The reaction was developed with TMB substrate, stopped with 2 M H_2_SO_4_, and absorbance was read at 450 nm. The endpoint titer for each sample was defined as the reciprocal of the highest dilution yielding an OD_450_ greater than 2.1 times the mean OD_450_ of negative control samples (serum or IgY from unimmunized hens). This longitudinal monitoring identified the peak antibody response window (days 42–49 post-immunization) for large-scale egg collection to maximize IgY yield for subsequent purification and the piglet passive immunization trial.

### 2.5. Preparation, Purification, and Characterization of IgY

Based on the antibody level monitoring results, eggs collected around the peak response time point were used for large-scale IgY preparation. Yolks were carefully separated and rolled on filter paper to remove residual egg white. Two established precipitation methods, ammonium sulfate precipitation and polyethylene glycol (PEG-6000) precipitation, were employed and compared for efficiency and purity, starting from a common pretreatment step. For the pretreatment, yolks were diluted 1:9 (*v*/*v*) with cold deionized water. The pH of the mixture was adjusted to 5.0–5.2 using 0.1 N HCl, followed by incubation overnight at 4 °C. The precipitate was removed by centrifugation at 10,000× *g* for 30 min at 4 °C, and the supernatant containing water-soluble proteins, including IgY, was collected. For ammonium sulfate precipitation, solid ammonium sulfate was slowly added to the supernatant to achieve 33% saturation. The solution was stirred for 1 h at 4 °C and centrifuged. The supernatant was further brought to 50% saturation with ammonium sulfate. The resulting precipitate, enriched with IgY, was collected by centrifugation. For PEG-6000 precipitation, solid PEG-6000 was added directly to the pretreated supernatant to a final concentration of 12% (*w*/*v*). The mixture was stirred for 1 h at room temperature and centrifuged, and the pellet was discarded. PEG-6000 was then added to the supernatant to a final concentration of 25% (*w*/*v*) to precipitate the IgY. The pellet was collected by centrifugation. After ammonium sulfate or PEG-6000 precipitation, the collected IgY pellets were dissolved in phosphate-buffered saline (PBS, pH 7.4). The solutions were then dialyzed using cellulose ester dialysis tubing with a molecular weight cut-off (MWCO) of 60–80 kDa (Biosharp, Beijing, China). The tubing was pre-boiled in deionized water for 10 min and rinsed thoroughly. The IgY solution was loaded into the tubing, leaving sufficient space for buffer exchange, and sealed at both ends. The dialysis bag was immersed in 2 L of PBS (pH 7.4) at 4 °C and stirred gently with a magnetic stirrer. The buffer was replaced with fresh PBS every 6–8 h, and dialysis was continued for 48 h (total of 6–8 buffer changes). After dialysis, the IgY solution was collected, centrifuged at 10,000× *g* for 15 min to remove any precipitated material, and the supernatant was stored at −20 °C.

The protein concentration of the purified IgY preparations from both methods was determined using a BCA Protein Assay Kit (Beyotime, Shanghai, China) according to the manufacturer’s instructions. A standard curve was generated using BSA (Solarbio, Beijing, China), and absorbance was measured at 562 nm to calculate and compare the IgY yields. The purity of the IgY preparations was subsequently assessed by SDS-PAGE and densitometric analysis. Samples (10 µg per lane) were separated under reducing conditions on 10% polyacrylamide gels and stained with Coomassie Brilliant Blue R-250. Gel images were analyzed using ImageJ software 1.54 (National Institutes of Health, Bethesda, MD, USA), and purity was calculated as the ratio of the combined grayscale intensity of the IgY heavy-chain (~68 kDa) and light-chain (~25 kDa) bands to the total intensity of all protein bands within the same lane. To confirm the antigen-specific reactivity of the purified antibodies, a Western blot analysis was performed. Vero cells were infected with PEDV-NX-2022 at a multiplicity of infection (MOI) of 0.1 for 24 h. Cells were washed three times with ice-cold PBS and lysed in RIPA buffer (Beyotime, Shanghai, China) containing protease inhibitor cocktail on ice for 30 min. The lysate was centrifuged at 12,000× *g* for 15 min at 4 °C, and the supernatant was collected. Protein concentration was determined using a BCA Protein Assay Kit (Beyotime, Shanghai, China) and adjusted to 5 μg/μL with loading buffer. An appropriate volume of 5× SDS loading buffer was added, and the mixture was denatured at 100 °C for 5 min. Denatured proteins were separated by SDS-PAGE and transferred onto a PVDF membrane. The membrane was then probed with the purified IgY (1:1500 dilution) to detect PEDV S protein epitopes, followed by a horseradish peroxidase (HRP)-conjugated goat anti-chicken IgY secondary antibody (1:5000 dilution; Abmart, Shanghai, China). To confirm equal protein loading, the membrane was also probed with a mouse anti-β-tubulin monoclonal antibody (1:2000 dilution; Abmart, Shanghai, China), followed by an HRP-conjugated goat anti-mouse IgG secondary antibody (1:5000 dilution; Abmart, Shanghai, China). Specific binding was detected using an ECL substrate (MedChemExpress, USA). The functional neutralizing activity of the purified IgY was determined by a virus neutralization test (VNT). Serial two-fold dilutions of the IgY preparation were mixed with an equal volume of the PEDV-NX-2022 strain containing 100 TCID_50_ and incubated at 37 °C for 1 h. These mixtures were then inoculated onto confluent Vero cell monolayers in 96-well plates. Appropriate virus-only and cell-only controls were included. The plates were incubated at 37 °C with 5% CO_2_ and observed daily for CPE over 5–7 days. The neutralization titer (NT_50_) was calculated as the reciprocal of the highest antibody dilution that protected 50% of the wells from CPE, using the Reed–Muench method.

### 2.6. Passive Immunization and Challenge Experiment in Piglets

Eighteen 4-day-old crossbred (Duroc × Landrace × Yorkshire) piglets, confirmed to be PEDV-naïve, were used in this study. After one day of acclimatization, the piglets were randomly assigned to three groups (n = 6 per group). The sample size of six piglets per group was chosen based on sample sizes commonly used in published PEDV challenge studies [10,11] and ethical considerations to minimize animal use. Group 1, designated the Treated Group (TG), consisted of piglets challenged with PEDV and treated with IgY purified from hens immunized with the recombinant adenovirus rADM-IFN-G-ped. Group 2, the Challenge Control (CC) group, included piglets challenged with PEDV and treated with IgY purified from hens immunized with the negative control adenovirus, rADM-blank. Group 3 served as the Blank Control (BC) group, which was not challenged and received an equivalent volume of phosphate-buffered saline (PBS). At five days of age, piglets in the TG and CC groups were orally inoculated with 1 × 10^5^ TCID_50_ of the PEDV-NX-2022 challenge strain. The dose of 20 mg total IgY per administration was selected based on previous studies showing that 10–30 mg of specific IgY provides effective passive protection against enteric viral infections in neonatal piglets [10,11]. The administration schedule (starting at 12 h post-challenge and continuing every 12 h for five doses) was designed to ensure the presence of neutralizing antibodies in the intestinal lumen before and during the peak phase of viral replication, which typically occurs between 24 and 72 h post-challenge [32]. All in vivo experiments were performed using a single, large-scale batch of IgY purified from eggs collected during the peak antibody response window (days 42–49 post-immunization). This batch was characterized by SDS-PAGE, densitometric analysis, BCA assay, Western blot, and virus neutralization test to ensure consistency and quality. All piglets were monitored for 14 days post-challenge. Survival was recorded daily from 0 to 7 days post-challenge for subsequent Kaplan–Meier survival curve analysis. Rectal swabs or fecal samples were collected daily during this period for the quantification of viral RNA. At the experimental endpoint—defined as death or euthanasia at 14 days post-challenge—a necropsy was performed on each piglet. Tissue segments from the duodenum, jejunum, and ileum were collected and fixed in 4% paraformaldehyde for histopathological examination.

### 2.7. Viral Load Quantification and Histopathological Analysis

Viral RNA was extracted from daily collected fecal samples using the QIAamp Viral RNA Mini Kit (QIAGEN, Hilden, Germany) according to the manufacturer’s instructions. The extracted RNA was used to quantify PEDV viral load via quantitative RT-qPCR using a method established by Zheng et al. (2018) [33]. The reaction employed specific primers targeting the PEDV N gene: forward primer 5′-AGTACGGGGCTCTAGTGCAG-3′ and reverse primer 5′- GCTTATCCAAATTCTTCAGGCG-3′. All reactions were performed on an Applied Biosystems 7500 Real-Time PCR System (Thermo Fisher Scientific, Waltham, MA, USA). Results were calculated and expressed as log_10_ viral RNA copies per gram of feces (log_10_ RNA copies/g). For histopathological examination, the fixed intestinal tissue samples (duodenum, jejunum, and ileum) were processed through a standard dehydration and clearing series, embedded in paraffin, and sectioned at a thickness of 4–5 µm. The tissue sections were then stained with hematoxylin and eosin (H&E) for microscopic evaluation of pathological changes. Histological images were captured using a Nikon Eclipse Ci-L upright light microscope (Nikon Corporation, Tokyo, Japan) at 40× magnification.

### 2.8. Statistical Analysis

All statistical analyses were performed using GraphPad Prism software (version 9.0). Before applying parametric tests, normality of data distribution was assessed using the Shapiro–Wilk test, and homogeneity of variances was assessed using Levene’s test. All quantitative data met the assumptions for parametric analysis (*p* > 0.05 for both tests in all groups). Survival data were analyzed by the Kaplan–Meier method, and differences between groups were compared using the log-rank test. Quantitative data, including antibody titers and IgY purity, were analyzed using one-way ANOVA followed by Tukey’s multiple comparisons test. For fecal viral load data, two-way ANOVA was performed followed by Šidák’s multiple comparisons test to compare the specific IgY and control IgY groups across days 1–3. All data are presented as the mean ± SEM. A *p*-value of less than 0.05 (*p* < 0.05) was considered statistically significant.

## 3. Results

### 3.1. Construction and Expression Analysis of the Recombinant Adenovirus rADM-IFN-G-Ped

**Figure 1:**
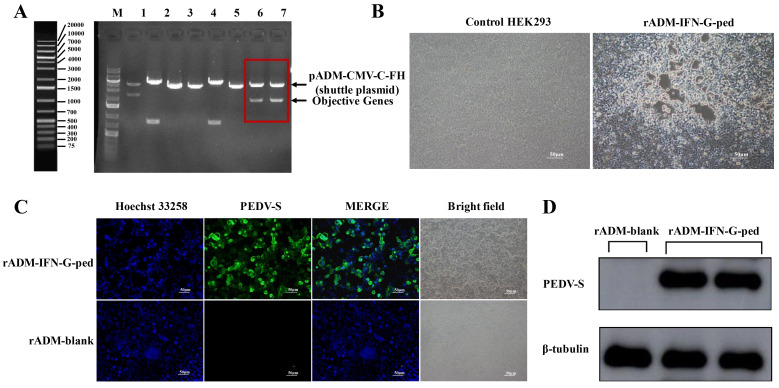
Construction and characterization of the recombinant adenovirus rADM-IFN-G-ped. (**A**) Restriction enzyme analysis of the recombinant shuttle plasmid pADM-CMV-C-FH-IFN-G-ped. Digestion with AsiSI and MluI yielded two fragments of approximately 1.8 kbp and 5.0 kbp, corresponding to the inserted gene and the linearized vector, respectively. (**B**) Rescue of rADM-IFN-G-ped in HEK 293 cells. The right panel shows adenovirus-induced CPE, while the left panel displays uninfected normal cells. (**C**) IFA detecting the expression of the S protein antigenic regions in HEK 293 cells infected with rADM-IFN-G-ped. (**D**) Western blot analysis confirming the expression of the S protein antigenic regions in HEK 293 cells infected with rADM-IFN-G-ped.

The codon-optimized ChIFN-γ-PEDV-S gene construct was cloned into the adenoviral shuttle plasmid pADM-CMV-C-FH. Restriction enzyme digestion of the resultant recombinant plasmid, pADM-CMV-C-FH-IFN-G-ped, with *AsiSI* and MluI yielded two distinct fragments of approximately 1.8 kbp and 5.0 kbp (Figure 1A), corresponding to the expected sizes of the inserted gene and the linearized vector backbone, respectively. This confirmed the successful construction of the recombinant shuttle plasmid. The linearized pADM-CMV-C-FH-IFN-G-ped plasmid was then co-transfected with the Ad5 backbone plasmid into HEK 293 cells to rescue the recombinant adenovirus, designated rADM-IFN-G-ped. A clear CPE, characterized by cell rounding and detachment, was observed 7 days post-transfection, whereas no CPE was evident in control wells (Figure 1B). Successful expression of the S protein antigenic regions was verified through complementary assays. An IFA using an anti-PEDV S protein monoclonal antibody revealed specific green fluorescent signals in rADM-IFN-G-ped-infected cells, which were absent in cells infected with the control adenovirus, rADM-blank (Figure 1C). This finding was corroborated by Western blot analysis. The same monoclonal antibody specifically recognized a band corresponding to the expected molecular weight in lysates of rADM-IFN-G-ped-infected HEK 293 cells, but not in lysates of rADM-blank-infected cells. A β-tubulin band was detected as a loading control, confirming equal protein loading (Figure 1D). Collectively, these results demonstrate the successful rescue of the recombinant adenovirus rADM-IFN-G-ped and its efficient expression of the PEDV S protein epitopes in mammalian cells.

### 3.2. Evaluation and Selection of the Optimal Method for IgY Purification

**Figure 2:**
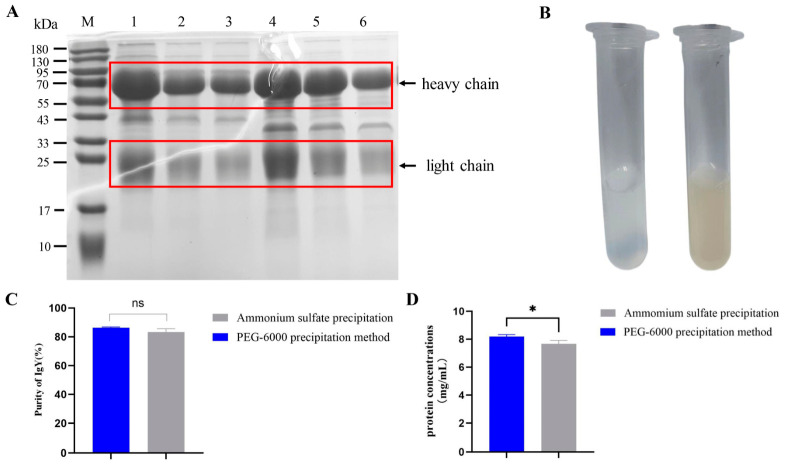
Comparison of two egg yolk antibody (IgY) purification methods. (**A**) SDS-PAGE analysis of IgY purified by ammonium sulfate precipitation (lanes 1–3) and PEG-6000 precipitation (lanes 4–6). Each preparation was loaded at three dilutions (5×, 10×, and 20×). The positions of the IgY heavy chain (HC, ~68 kDa) and light chain (LC, ~25 kDa) are indicated. (**B**) Macroscopic appearance of the final purified products: PEG-6000-precipitated IgY (left) and ammonium sulfate-precipitated IgY (right). (**C**) Quantitative purity comparison of the two methods based on densitometric analysis using ImageJ. Purity was calculated as the percentage of total lane intensity contributed by the IgY HC and LC bands. Data are presented as mean ± SEM; n = 3 independent purifications. (**D**) Protein concentration of the final purified IgY preparations as determined by BCA assay. Data are presented as mean ± SEM; n = 3 independent measurements. *, *p* < 0.05; ns, no significance (Student’s *t*-test).

The water-soluble fractions of IgY, purified using two distinct methods (ammonium sulfate precipitation and PEG-6000 precipitation), were analyzed by SDS-PAGE. Both methods yielded preparations exhibiting prominent bands at approximately 68 kDa and 25 kDa (Figure 2A), corresponding to the heavy and light chains of IgY, respectively. A qualitative visual assessment of the final products indicated that the PEG-6000 preparation was homogeneous, semi-transparent, and pale grayish-white, whereas the ammonium sulfate preparation appeared more turbid and opaque with a yellowish tint (Figure 2B), suggesting lower residual lipid/protein impurities in the former. Quantitative densitometric analysis of the Coomassie-stained gels using ImageJ software was performed to compare purity objectively. The calculated purity was 83.3% for the ammonium sulfate method and 86.3% for the PEG-6000 method (Figure 2C). Furthermore, the protein concentration of the final purified products, as determined by BCA assay, was significantly higher for the PEG-6000 method (8.18 mg/mL) compared to the ammonium sulfate method (7.67 mg/mL) (Figure 2D). In summary, based on superior purity, higher final protein concentration, and favorable physical characteristics indicating fewer co-precipitating impurities, the PEG-6000 precipitation method was selected for all subsequent large-scale IgY purification in this study.

### 3.3. Antibody Response in Immunized Hens and Neutralizing Activity of IgY

**Figure 3:**
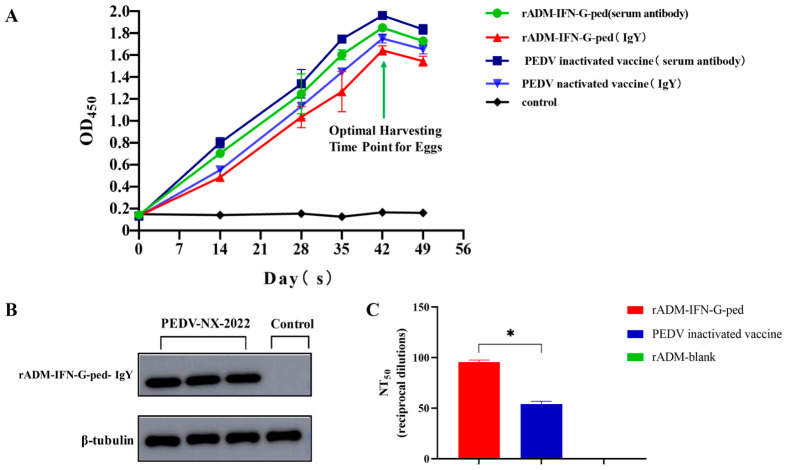
Antibody response in immunized hens and characterization of IgY. (**A**) Dynamic changes in PEDV-specific antibody levels measured by indirect ELISA. Serum samples were tested at a 1:400 dilution, and yolk IgY samples at a 1:200 dilution, based on preliminary exper-iments to ensure OD_450_ values within the linear range. Each point represents the mean OD_450_ ± SEM of six biological replicates (n = 6). The green arrow indicates the optimal egg collection win-dow. The endpoint titers (calculated as described in Section 2.4) were used to confirm the antibody response dynamics, while the figure shows the raw OD_450_ kinetics for clarity. (**B**) Western blot analysis demonstrating the specificity of IgY purified from hens immunized with rADM-IFN-G-ped. Purified PEDV virions were probed with the IgY preparation. (**C**) Virus neutralization titers (VNT, expressed as NT_50_) of purified IgY from hens immunized with rADM-IFN-G-ped or the commercial vaccine. Data are presented as mean ± SEM; *, *p* < 0.05 (Student’s *t*-test).

The dynamic profile of PEDV-specific antibodies in serum and egg yolk from immunized hens was monitored by ELISA (Figure 3A). In Figure 3A, each data point represents the mean OD_450_ value (±SEM) measured at a fixed serum dilution of 1:400 or yolk IgY dilution of 1:200. Following the primary immunization with the recombinant adenovirus rADM-IFN-G-ped, serum antibody levels rose rapidly within the first two weeks. Yolk antibody levels lagged behind, remaining low until after the second booster. Both serum and yolk antibody levels peaked during the second week after the third immunization (days 42–49 post-primary immunization), identifying this period as the optimal window for egg collection to maximize IgY yield. Although hens immunized with the commercial inactivated PEDV vaccine consistently showed slightly higher OD_450_ values than the rADM-IFN-G-ped group, the overall kinetic patterns were similar, with yolk OD_450_ values generally lower than corresponding serum values in both groups. Endpoint titers (calculated as described in Section 2.4) followed the same kinetic trend and confirmed the peak response period. Western blot analysis confirmed the specificity of the induced antibodies. IgY purified from hens immunized with rADM-IFN-G-ped specifically recognized the PEDV-NX-2022 viral antigens (Figure 3B). Despite the modestly lower ELISA OD values, the functional neutralizing activity of the IgY was superior. VNT revealed that the IgY from the rADM-IFN-G-ped group had a significantly higher neutralization titer (1:96) compared to the IgY from the commercial vaccine group (1:52) (Figure 3C).

### 3.4. Passive Immunization with IgY Protects Piglets from Lethal PEDV Challenge

**Figure 4:**
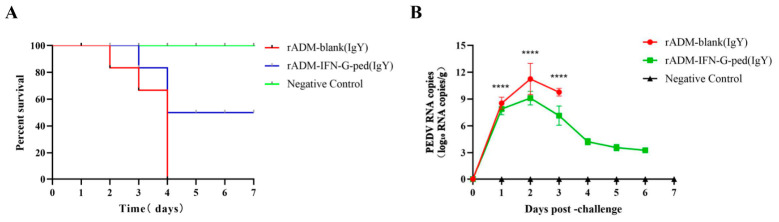
Passive immunization with IgY improves survival and reduces viral load in PEDV-challenged piglets. (**A**) Kaplan–Meier survival curves of piglets in different groups following PEDV challenge. (**B**) Daily fecal viral shedding, expressed as log_10_ PEDV RNA copies per gram, measured by RT-qPCR from day 0 to day 7 post-challenge. Data are presented as mean ± SEM. Statistical comparisons between the Specific IgY and Control IgY groups across days 1–3 were performed using two-way ANOVA with Šidák’s multiple comparisons test (****, *p* < 0.0001).

The protective efficacy of IgY (purified from hens immunized with rADM-IFN-G-ped) was evaluated in a passive immunization-challenge model using 5-day-old suckling piglets. Following oral challenge with the virulent PEDV-NX-2022 strain, piglets treated with specific IgY showed significantly improved outcomes compared to those receiving control IgY (from hens immunized with rADM-blank). A stark difference in survival was observed (Table 1). The specific IgY treatment group achieved a 50% survival rate (3/6), whereas all piglets (0/6) in the control IgY group succumbed to infection. Unchallenged control piglets remained healthy throughout the study. Kaplan–Meier survival analysis (Figure 4A) detailed the mortality timeline: all deaths in the control IgY group occurred between days 2 and 4 post-challenge, while in the specific IgY group, mortality was limited to 3 piglets, with deaths occurring on days 3 and 4. Virological monitoring by RT-qPCR revealed that oral administration of specific IgY effectively suppressed intestinal viral replication. While fecal viral shedding peaked around day 2 in all challenged animals, the mean viral load on days 1, 2, and 3 post-challenge was significantly higher in the control IgY group compared to the specific IgY treatment group (Figure 4B). This early reduction in viral shedding correlated with a quicker resolution of clinical diarrhea. These results demonstrate that IgY targeting the PEDV S protein epitopes can neutralize the virus within the intestinal tract, thereby reducing viral replication, attenuating disease severity, and providing significant protection against lethal PEDV challenge in neonatal piglets.

### 3.5. Histopathological Analysis

**Figure 5:**
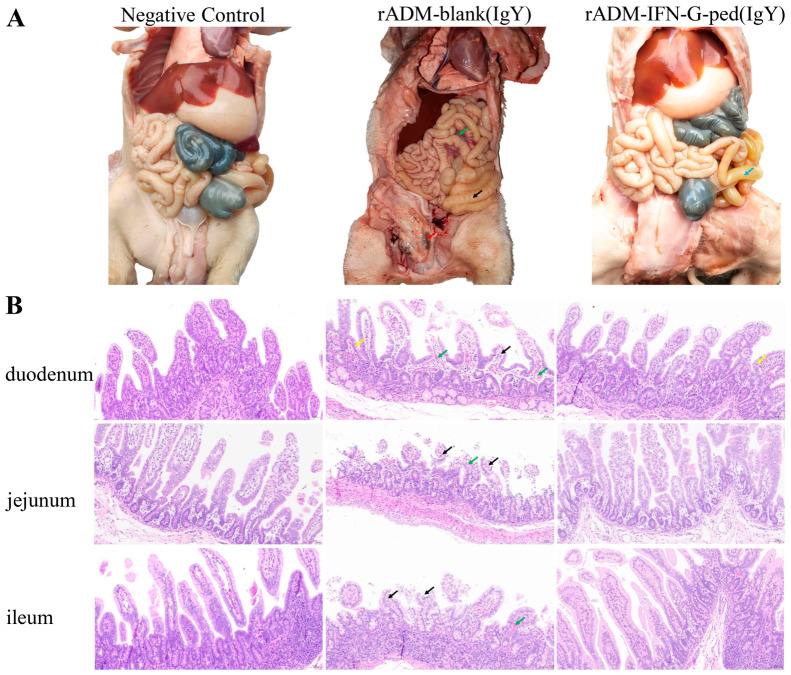
Histopathological analysis of intestinal tissues. (**A**) Representative gross lesions observed during necropsy of piglets from different treatment groups. (**B**) Histological sections of duodenum, jejunum, and ileum stained with H&E. Images were captured using a Nikon Eclipse Ci-L upright light microscope (Nikon Corporation, Tokyo, Japan) at 40× magnification.

Gross and microscopic examinations revealed significant intestinal damage in PEDV-challenged piglets, which was markedly attenuated by treatment with specific IgY. Representative necropsy findings are shown in Figure 5A. Compared to the normal intestinal morphology of unchallenged controls, piglets treated with control IgY (rADM-blank group) exhibited severe intestinal distension, thin intestinal walls with undigested milk clots (black arrow), congested mesenteric capillaries (green arrow), and edema of the mesenteric and inguinal lymph nodes (red arrow). In contrast, piglets treated with specific IgY (rADM-IFN-G-ped group) showed substantially restored intestinal integrity, with only mild fluid accumulation in the ileum (blue arrow). Histopathological analysis of H&E-stained intestinal sections further detailed these findings (Figure 5B). In the duodenum, tissues from the control IgY group showed villous blunting (black arrow), a marked reduction in goblet cells (yellow arrow), and capillary congestion in the lamina propria (green arrow) compared to the intact villous structure of unchallenged controls. The specific IgY group exhibited restored villous architecture with abundant goblet cells (yellow arrow). In the jejunum, the control IgY group displayed severe villous atrophy and epithelial denudation (black arrow) and capillary congestion (green arrow). The specific IgY group maintained well-developed, intact villi without significant necrosis or inflammatory infiltration. In the ileum, the control IgY group showed focal villous shortening (black arrow) and capillary congestion (green arrow). The specific IgY group presented preserved villous structure and epithelial integrity. Collectively, these results demonstrate that treatment with PEDV-specific IgY significantly alleviated the characteristic gross and histopathological lesions induced by PEDV infection, preserving intestinal mucosal integrity across all examined segments.

## 4. Discussion

Current control strategies relying on sow vaccination often fail to induce sufficient mucosal immunity, leaving piglets vulnerable. In this study, we demonstrate that a novel recombinant adenovirus vaccine, rADM-IFN-G-ped, effectively stimulates neutralizing IgY in hens. Oral administration of these antibodies conferred significant protection against lethal PEDV-NX-2022 challenge in neonatal piglets, as evidenced by improved survival, reduced viral shedding, and alleviated intestinal pathology.

The PEDV spike glycoprotein plays a dual role in establishing infection and inducing neutralizing antibodies [34], establishing it as a critical target for the rational design of both live-attenuated and subunit vaccines [35]. However, concerns have been raised that vaccines employing the full-length S protein might potentially induce adverse immune responses or off-target effects [36]. Notably, Thavorasak et al. (2022) [37] identified an enhancing epitope within the S protein using phage display technology and a specific monoclonal antibody (mAbG3). This antibody binds to multiple discontinuous epitopes within the S1-0 subdomain of the S1 subunit and, unexpectedly, enhances PEDV entry into susceptible Vero cells, which lack Fc receptors. To mitigate such concerns, our vaccine strategy deliberately employed a concatenated construct comprising four selected B-cell epitope regions from the PEDV S protein, rather than the full-length protein. This multi-epitope approach was designed to focus the immune response on key neutralizing domains while potentially avoiding immunodominant but non-neutralizing or poorly neutralizing regions that might otherwise divert or interfere with the generation of protective immunity. Neutralizing antibodies confer protection by directly binding to critical pathogen structures—such as viral surface glycoproteins—thereby blocking host cell entry and subsequent infection [38]. Consequently, the neutralizing antibody titer is widely regarded as one of the “gold standard” correlates of vaccine immunogenicity [39]. Although hens immunized with the commercial inactivated PEDV vaccine exhibited modestly higher total IgY titers as measured by ELISA, the IgY derived from our recombinant adenovirus vaccine group demonstrated a significantly higher neutralizing titer (NT_50_ 1:96 versus 1:52). While this observation is encouraging and aligns with the intended goal of focusing on the immune response, we caution against interpreting this as definitive evidence of functional superiority over alternative antigen designs. Our study lacked a head-to-head comparator group immunized with a full-length S protein expressed in the same adenoviral backbone. Consequently, the enhanced neutralization could be attributed to the multi-epitope design [40], the co-expression of ChIFN-γ [41], or intrinsic properties of the adenoviral vector platform [42]. Nevertheless, these findings confirm that the multi-epitope construct is immunogenic and capable of eliciting potent neutralizing antibodies, thereby validating the feasibility of this specific design for passive immunization.

Our in vivo findings both corroborate and extend previous studies on passive immunization against PEDV using IgY. In the present study, all piglets in the control IgY-treated group succumbed to infection, whereas 50% of piglets in the specific IgY-treated group survived and recovered. This survival rate is marginally higher than the 49.24% reported by Kweon et al. (2000) [11] using IgY from inactivated whole virus, but considerably lower than the 100% protection reported by Lee et al. (2015) [10] using IgY raised against the recombinant S1 domain. We speculate that these discrepancies may be attributable to differences in antigen design, IgY dose, administration regimen, or the virulence and challenge dose of the viral strain employed [43,44]. Critically, the significant reduction in fecal viral load observed in the treatment group from days 1 to 3 post-challenge provides direct virological evidence that orally administered IgY can neutralize virus within the intestinal lumen, thereby interrupting viral replication and reducing environmental contamination. This virological efficacy underpins the observed clinical improvements and survival benefit.

Several limitations of this study should be acknowledged. First, the challenge experiment employed a single G2b strain (PEDV-NX-2022); given the substantial genetic and antigenic diversity of the PEDV S protein across G1, G2a, G2b, and S-INDEL lineages [45], the cross-protective efficacy of this IgY against heterologous strains remains untested, and even within G2b, field strain variability may limit generalizability [46]. Second, the conceptual advance of the multi-epitope design remains inferential, as the study lacks a direct head-to-head comparison with IgY raised against full-length S protein or alternative antigen formats delivered via the same adenoviral backbone. Consequently, the specific contribution of the multi-epitope architecture cannot be definitively quantified. Third, although the sample size (n = 6 per group) was sufficient to detect a statistically significant survival difference, larger-scale studies are required to confirm protective efficacy and assess field variability. Fourth, the IgY administration protocol used here (20 mg per dose, five doses over 60 h) was designed for maximum efficacy in a controlled ex-perimental setting; future work should establish minimal effective dosing regimens, simplified schedules, and practical delivery methods suitable for field application. In conclusion, this study provides proof-of-concept for the feasibility of a complementary passive immunization strategy against PEDV using adenovirus-vectored multi-epitope IgY, highlighting the potential of the hen-IgY platform for rapid and scalable antibody production.

## 5. Conclusions

In this study, we successfully constructed a novel recombinant adenovirus, rADM-IFN-G-ped, co-expressing four key neutralizing epitope regions of the PEDV G2b S protein and chicken IFN-γ as a molecular adjuvant. Immunization of laying hens with this construct elicited robust PEDV-specific humoral immune responses, enabling the production of high-titer IgY. The purified IgY demonstrated potent virus-neutralizing activity in vitro, with an NT_50_ titer (1:96) significantly exceeding that of IgY derived from a commercial inactivated vaccine (1:52). In a passive immunization-challenge model using neonatal piglets (n = 6 per group), oral administration of this specific IgY was associated with improved clinical outcomes, including a 50% survival rate compared to 0% in the control group, and significantly reduced fecal viral shedding during the early phase of infection. Histopathological examination further indicated that IgY treatment alleviated PEDV-induced intestinal lesions, including villous atrophy, epithelial necrosis, and inflammatory infiltration. Collectively, these preliminary findings provide proof-of-concept that the hen-adenovirus-IgY platform represents a feasible strategy for generating pathogen-specific antibodies against PEDV. However, given the modest sample size and the controlled experimental conditions, these results should be interpreted with caution and regarded as an initial step toward validation. The IgY dose and administration schedule employed here offer a foundation for future optimization, but further studies with larger sample sizes and diverse field conditions are warranted to confirm the protective efficacy and to assess the broader applicability of this approach in neonatal piglets.

## Figures and Tables

**Table 1:** Statistics of the incidence of piglets in each group.

Parameters	rADM-IFN-G-Ped Recombinant Adenovirus IgY Treatment Group	rADM-Blank Negative Control Adenovirus IgY Treatment Group	Negative Control Group
Sample Size	6	6	6
Number of Cases	6	6	0
Number of Recovered Cases	3	0	-
Number of Deaths	3	6	-
Mortality Rate (%)	50	100	0
Recovery Rate (%)	50	0	-

## Data Availability

The original contributions presented in this study are included in the article. Further inquiries can be directed to the corresponding author.

## Abbreviations

The following abbreviations are used in this manuscript:

PEDV	Porcine epidemic diarrhea virus
PED	Porcine epidemic diarrhea
IgY	Egg yolk antibodies
sIgA	Secretory immunoglobulin A
Ad5	Adenovirus serotype 5
